# Lipid and volatile dynamics shape sweet potato and honey aroma in ‘Baiye 1’ black tea

**DOI:** 10.1016/j.fochx.2025.102823

**Published:** 2025-07-21

**Authors:** Lin Chen, Yuxuan Shi, Jingyi Pan, Yueling Zhao, Liping Liu, Qun Ye, Yuefei Wang, Zhonghua Liu, Ping Xu

**Affiliations:** a Institute of Tea Science, Zhejiang University, Hangzhou 310058, China; b Department of Tea Science, Sichuan Agricultural University, Chengdu 611130, China; c Huzhou Academy of Agricultural Sciences, Huzhou 313099, China; d Provincial Key Agricultural Enterprise Research Institute of Mingbao Natural Plant Extracts, Hangzhou 310020, China; e Key Laboratory of Tea Science of Ministry of Education, Hunan Agricultural University, Changsha 410128, China

**Keywords:** Albino tea cultivar, Black tea, Amino acid, Lipid transformations, Flavor quality

## Abstract

The albino tea cultivars represented by ‘Baiye 1’, characterized by their naturally enriched amino acid content and reduced polyphenol levels, exhibit distinctive sweet and umami flavors along with honey-like aromas, characteristics that are uncommon in traditional black teas. Nevertheless, the metabolic transformations underlying these superior sensory qualities during processing remain insufficiently understood. Here, UPLC-HRMS and HS-SPME-GC–MS/MS were employed to analyze non-volatile and volatile compounds respectively during ‘Baiye 1’ black tea (BYBT) processing. Six non-volatile compounds were identified as key taste components responsible for BYBT's sweet, umami, and mild flavors, while eight aroma-active volatiles, including β-ionone and geraniol, collectively shaped BYBT's baked sweet potato and honey-like aroma. Interestingly, lipidomics and aroma formation pathway analysis revealed that lipid transformations during rolling and drying released precursors and generated key aroma components, highlighting the pivotal role of lipid metabolism in aroma formation. Our work elucidates the flavor development mechanisms of high-amino-acid black tea and offers biochemical insights for optimizing processing and enhancing the value of specialty cultivars like ‘Baiye 1’.

## Highlights

• Sweetness (L-Met, L-Ser, L-Pro) and umami (L-Asn, GABA) markers enhance BYBT taste.
• Eight VOCs identified as key contributors to sweet potato and honey-like aromas.
• Rolling increases AGs and GPLs, degrades GLs, and shapes BYBT flavor.
• Lipid oxidation and Maillard reactions drive BYBT aroma formation pathways.

## Graphical abstract

**Figure:**
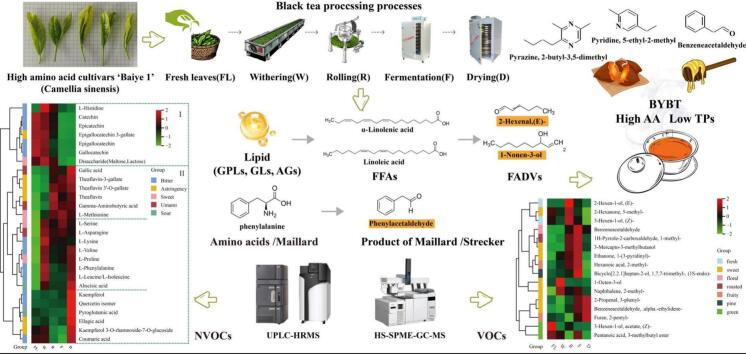

## 1 Introduction

Black tea, the most widely consumed tea globally, is traditionally produced from tea cultivars with high polyphenol content, as these compounds are essential for developing the characteristic strong flavor and deep color during fermentation (Aaqil et al., 2023; Zhang et al., 2019). However, excessive polyphenols often result in undesirable bitterness, limiting consumer acceptance and restricting its application in flavored food products (Ye et al., 2022). According to the carbon‑nitrogen balance mechanism in plants, elevated levels of tea polyphenols are typically inversely correlated with amino acid content (Wang, Wang, et al., 2021). This trade-off poses a significant challenge in balancing flavor intensity and sensory quality in black tea production.

Extensive research has demonstrated that amino acids in tea leaves not only contribute directly to umami and sweetness but also participate in Maillard reactions and Strecker degradation, generating critical aroma compounds, such as phenylacetaldehyde (with honey-like notes) and heterocyclic compounds (with nutty and roasted notes) (Ho et al., 2015; Yang et al., 2022). Nevertheless, current understanding remains predominantly limited to high-polyphenol cultivars, where amino acid metabolism is largely governed by polyphenol oxidation pathways. This frequently results in excessive accumulation of Strecker aldehydes, like 3-methylbutanal, that overwhelm more subtle flavor nuances (Chen et al., 2021). ‘Baiye 1’ (*Camellia sinensis*), an albino tea cultivar characterized by high natural amino acid and low polyphenol content, presents a promising option for diversifying tea products and promoting sustainable development within the industry (Saleem et al., 2024; Zhu et al., 2024). This biochemical signature is a hallmark of albino phenotypes, which typically exhibit impaired chloroplast development that leads to metabolic reprogramming, favoring the accumulation of nitrogen-containing compounds (especially free amino acids) at the expense of carbon-based secondary metabolites (such as polyphenols) (Yang et al., 2022). Although the biochemistry of albino green tea is partially understood, the metabolic consequences of subjecting this unique raw material to the strong oxidative and thermal stress of black tea processing remain largely unknown. Our recent analysis of the flavor and compositional characteristics of the seasonal ‘Baiye 1’ black tea (BYBT) revealed its unique sensory profile. Compared to black teas produced from alternative varieties, BYBT exhibits a polyphenol content approximately 30 % lower and an amino acid content approximately 20 % higher, contributing to reduced bitterness and enhanced umami. Furthermore, BYBT displays a distinctive aroma reminiscent of sweet potato and honey, attributed to the accumulation of compounds such as β-ionone (Chen et al., 2025; Zhang et al., 2023). However, the dynamic changes during its formation that underlie these specific flavor characteristics remain unclear.

In the present work, we characterized qualitative and quantitative changes in non-volatile metabolites throughout four consecutive processing steps using UPLC-HRMS, while tracking volatile compound dynamics across these stages with HS-SPME-GC–MS/MS. Multivariate statistical analysis and random forest (RF) methods were utilized to identify key differential metabolites, while relative odor activity value (rOAV) analysis was applied to determine the key aroma compounds in the finished black tea and explore their potential formation pathways. The findings provide unprecedented insights into the biochemical mechanisms of high-amino acid tea processing. They offer valuable guidance for optimizing production techniques and developing novel black teas with low bitterness, mild astringency, and rich, sweet aromas to cater to the preferences of younger consumers.

## 2 Materials and methods

### 2.1 Chemicals and materials

**Fig. 1:**
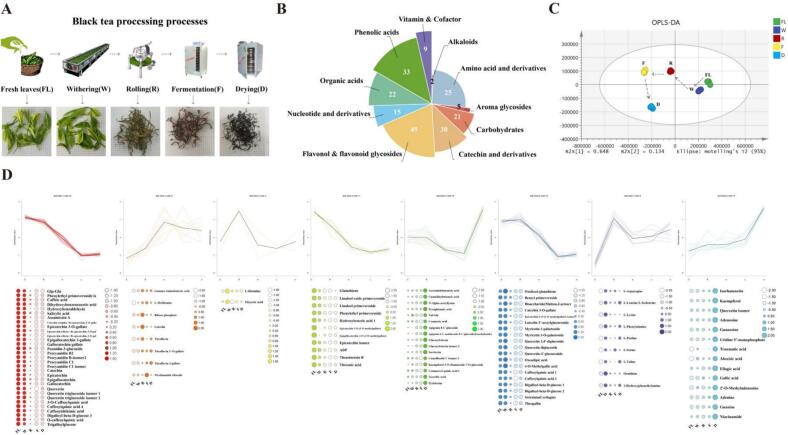
Dynamic changes of NVOCs during processing stages of BYBT. (A) Schematic diagram of processing stages for black tea, (B) Classification of NVOCs, (C) Dynamic trajectory of NVOCs, (D) K-means clustering of differential NVOCs.

Methanol, acetonitrile, and formic acid used for mass spectrometry analysis were purchased from Fisher Scientific (Fair Lawn, NJ, USA). Mass spectrometry-grade ammonium bicarbonate and chromatography-grade methyl tert-butyl ether were sourced from Sigma Aldrich (St. Louis, MO, USA), and chromatography-grade hexane was obtained from Merck (Darmstadt, Germany). Ultrapure water was prepared using the Merck Milli-Q water purification system. The gas chromatography–mass spectrometry (GC–MS/MS) system used was an Agilent 8890-7000E, equipped with a DB-5MS column (30 m × 0.25 mm × 0.25 μm). Headspace solid-phase microextraction needles (120 μm DVB/CWR/PDMS) were purchased from Agilent (Santa Clara, CA, USA). On April 14, 2023, one bud with two leaves of ‘Baiye 1’ (*Camellia sinensis*) were harvested from the Lishui Institute of Agricultural and Forestry Sciences (119.94°N, 28.47°E) and processed into black tea. The process encompasses withering, rolling, fermentation and drying, employing standard black tea processing techniques and integrating the optimized parameters as established in our preceding research (Chen et al., 2021; Chen et al., 2025). The withering was conducted at 28–32 °C for 14–16 h until the leaves achieved a moisture content of 58–62 %, as monitored by a rapid moisture analyzer (≥ 3 replicates). Subsequently, withered leaves were rolled using a 6CR-35 roller (40 rpm) for 75 min to disrupt cellular structures, enabling enzymatic oxidation during fermentation. Rolled leaves were then fermented for 3 h at 32 °C and 90 % relative humidity. The drying process was initiated at 120 °C for 30 min, followed by 95 °C for 2 h. Samples from the fresh leaf (FL), withering (W), rolling (R), fermentation (F), and drying (D) stages were freeze-dried and stored at −20 °C until analysis (Fig. 1A). Samples were collected in triplicate at each processing stage and subjected to a two-day BYBT protocol to ensure reliability.

### 2.2 Detection of NVOCs

A metabolomics and lipidomics analysis platform previously reported (Du et al., 2022) was used. Briefly, tea samples were analyzed using the Meta-Phenotyper™ platform, a high-coverage, high-precision metabolomics platform. Three UHPLC-HRMS-based analytical methods were employed for comprehensive metabolite and lipid detection. Metabolomics analysis was conducted on an Ultimate™ 3000 UHPLC system coupled with a Q Exactive™ quadrupole-Orbitrap mass spectrometer (Thermo Scientific, San Jose, USA). The hydrophilic extract was analyzed on an ACE C18-PFP column (Advanced Chromatography Technologies Ltd., Aberdeen, Scotland) in positive electrospray mode and an Acquity HSS C18 column (Waters Corporation, Milford, USA) in negative electrospray mode. Lipidomics analysis was performed on the same instrument in positive/negative polarity switching mode, using an Accucore C30 core-shell column (Thermo Scientific, Bellefonte, USA). Full scan mass spectrometry data were acquired at 70,000 FWHM resolution, with Top 7 or Top 10 data-dependent MS/MS spectra. Data acquisition used XCalibur software (Thermo Scientific, San Jose, USA), and quality control (QC) samples were evenly inserted into each batch.

### 2.3 Detection of VOCs

Samples were immediately weighed, frozen in liquid nitrogen, and stored at −80 °C. They were ground into powder in liquid nitrogen, and 500 mg (1 mL) was transferred to a 20 mL headspace vial (Agilent, Palo Alto, CA, USA) containing a saturated NaCl solution to inhibit enzymatic reactions. The vial was sealed with a TFE‑silicone headspace septum (Agilent). For solid-phase microextraction (SPME), each vial was heated at 90 °C for 5 min, and a 120 μm DVB/CWR/PDMS fiber (Agilent) was exposed to the headspace for 15 min at 60 °C. VOCs were desorbed at 250 °C for 5 min in splitless mode using an Agilent Model 8890 gas chromatograph. Identification and quantification were performed using an Agilent 7000D mass spectrometer equipped with a DB-5MS capillary column (30 m × 0.25 mm × 0.25 μm). Helium was used as the carrier gas at 1.2 mL/min. The injector temperature was maintained at 250 °C, and the column temperature program was as follows: 40 °C (3.5 min), 10 °C/min to 100 °C, 7 °C/min to 180 °C, and 25 °C/min to 280 °C (5 min). Mass spectrometry was recorded in electron ionization (EI) mode at 70 eV, with quadrupole, ion source, and transfer line temperatures set to 150 °C, 230 °C, and 280 °C, respectively. The mass spectrometer operated in selected ion monitoring (SIM) mode for analyte identification and quantification. Volatile components were identified by comparing the obtained mass spectra with reference mass spectra from the National Institute of Standards and Technology (NIST) mass spectral library. The relative concentration of volatile compounds was expressed as the ratio of the peak area of each volatile component to that of the internal standard. The rOAV quantifies the contribution intensity of an aroma compound by calculating the ratio of its relative concentration to its odor detection threshold. Higher rOAV values indicate greater aroma impact. Odor threshold data are obtained from literature sources and established databases (VCF online).

### 2.4 Statistical and omics data analysis

Statistical analysis and data visualization were performed using the Metware Cloud platform (https://cloud.metware.cn), SIMCA-14.1, Origin 2024, TBtools-II (https://www.tbtools.com), Cytoscape 3.8.0, and Adobe Illustrator 2023. Group comparisons were based on VIP > 1, FC > 1 or ≤ 0.5, and *p* < 0.05. VIP values were extracted from OPLS-DA results, and permutation testing (200 iterations) was performed to prevent overfitting. Model predictive parameters (R^2^X, R^2^Y, Q^2^) were evaluated, with values closer to 1 indicating greater stability and reliability. A Q^2^ > 0.5 indicates a valid model, while Q^2^ > 0.9 suggests an excellent model. All experiments were conducted in triplicate, and data are presented as mean ± standard deviation.

## 3 Results and discussion

### 3.1 Dynamic changes in NVOCs during BYBT processing

To clarify the dynamic changes of taste-related metabolites during BYBT processing, NVOCs were analyzed using UPLC-HRMS. A total of 547 NVOCs were identified, including 296 lipids. The remaining 251 metabolites were classified into various categories as depicted in Fig. 1B. Given that lipid oxidation is a major pathway for aroma formation in tea processing (Zhang, Hua, et al., 2025), we subsequently investigated the correlation between the degradation of the identified lipid pools and the generation of key aroma compounds, as detailed in Section 3.5. Orthogonal Partial Least Squares Discriminant Analysis (OPLS-DA) was employed to analyze the data, with model parameters (R^2^Y = 0.997, Q^2^ = 0.96) indicating excellent fit and predictive capability (Fig. 1C). The OPLS-DA plot revealed a clear spatial distribution of metabolites across the processing stages, with samples from the FL stage in the first quadrant, W in the fourth, R and F in the second, and D in the third. This distribution highlighted significant metabolic shifts, particularly during the rolling and drying stages. This clear separation between processing stages underscores the dynamic nature of NVOCs during BYBT production. While our OPLS-DA model highlights rolling and particularly drying as periods of major metabolic shifts, other studies on black tea have emphasized the importance of earlier stages. For instance, Yang reported significant changes in non-volatile metabolites occurring during the withering phase (Yang et al., 2024), and Wu observed the most pronounced metabolic differences during both withering and rolling (Wu et al., 2019). Drying is the primary thermal processing step, inducing significant non-enzymatic reactions such as Maillard reactions, Strecker degradation, and thermal degradation or transformation of compounds like amino acids and glycosides (Wang, Chen, et al., 2024). Given the unique biochemical profile of ‘Baiye 1’ (high amino acid, low polyphenol), the impact of these thermal reactions during drying could be relatively more pronounced compared to the enzymatic changes dominating the earlier stages in traditional cultivars. Furthermore, the substantial water removal during drying concentrates metabolites and alters the reaction environment, contributing further to the significant metabolic shifts observed in this final stage (Wang, Liu, et al., 2024).

Differential metabolites (DAMs) were subsequently screened from the OPLS-DA model based on criteria of VIP ≥ 1, |log2(FC)| ≥ 2, and *p* ≤ 0.05. K-means clustering analysis of the 110 identified DAMs revealed eight distinct variation trends (Fig. 1D). These observed trends in metabolic changes were generally consistent with those reported in the literature for black tea processing (Wang, Chen, et al., 2021). For instance, 30 metabolites (categories 3, 6, and 7), reached their highest levels during W. This included the significant accumulation of amino acids, likely resulting from protein hydrolysis and stress-induced synthesis (Li et al., 2025a). During the withering process, the characteristic metabolic reprogramming in albino cultivars, resulting from their impaired chloroplast development, reportedly enhances amino acid biosynthesis while reducing polyphenol production (Ye et al., 2024). Another set of 30 metabolites (categories 5 and 8), primarily flavonols, flavonoid glycosides, and nucleotides and their derivatives (53.3 %), showed significant increases during the drying stage. Meanwhile, the dipeptide Val-Glu (Category 5), resulting from protein degradation during drying, is a reported umami peptide and potentially contributes to the umami sensation in BYBT (Kong et al., 2017).

### 3.2 Contribution of NVOC changes to BYBT taste characteristics

**Fig. 2:**
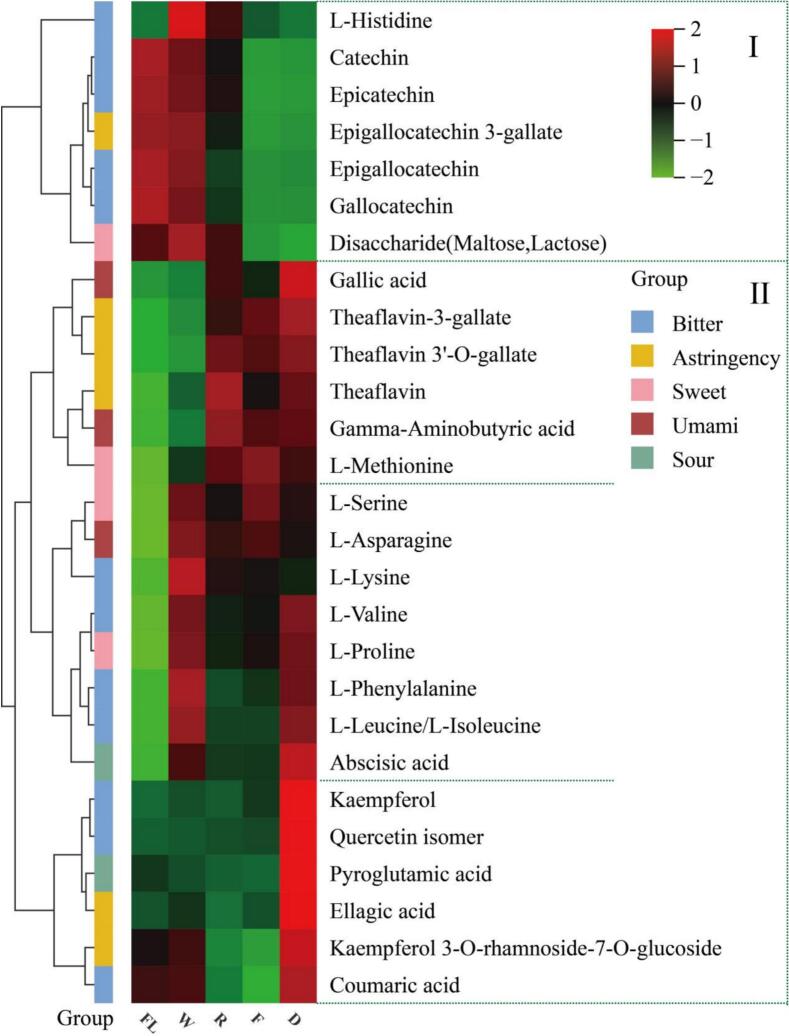
Dynamic changes of 27 key taste-related differential NVOCs during processing.

A heatmap was constructed to visualize the dynamic changes of 27 taste-associated differential metabolites during BYBT processing (Fig. 2). Hierarchical clustering divided these metabolites into two main groups. Cluster I primarily comprised non-gallated catechins (C, EC, GC, EGC), associated with bitterness, and gallated catechins (EGCG), linked to both bitterness and astringency. The decreasing trend of catechin content, particularly after rolling and fermentation, is a typical characteristic of black tea processing, mainly caused by enzymatic oxidation catalyzed by polyphenol oxidase (PPO) and peroxidase (POD) (Yassin et al., 2015). Further reductions during drying result from isomerization, degradation, polymerization, and interactions with L-theanine, glucose, and proteins (Wang, Liu, et al., 2024). Compared to traditional high-polyphenol cultivars, the lower initial catechin content of ‘Baiye 1’ implies that its final processed tea may have fewer residual substances contributing to bitterness and astringency, which likely contributes to its mellow taste. In the first subgroup, Theaflavin 3’-O-gallate increased by 20.48 % after drying compared to fresh leaves. These Theaflavins (TFs) contribute significantly to the tea's aftertaste and richness (Zhang, Yang, et al., 2020). Although the lower catechin substrate in ‘Baiye 1′ might limit the total amount of TFs, their relative contribution to the overall taste profile could be more prominent in this low-catechin context. Concurrent with the decrease in esterified catechins (ECG, EGCG), an increase in gallic acid was observed, indicating hydrolysis, which can help reduce astringency and balance taste (Yu et al., 2022).In the second subgroup, the accumulation of amino acids (e.g., l-serine, L-proline, L-asparagine, l-lysine) during withering provides processing evidence for the high amino acid characteristic of ‘Baiye 1′ and directly contributes to its umami and sweet taste. Although these accumulated amino acids subsequently act as precursors for aroma formation (e.g., through Strecker degradation) during fermentation and drying, their levels remain higher than those in the fresh leaves.The third subgroup included flavonols and their glycosides. The increasing pattern of these compounds during the drying process reflects their differences in relative thermal stability; this is consistent with literature reports that high temperatures (> 120 °C) can lead to their significant degradation, and Kaempferol is generally more heat-stable than quercetin (Xue et al., 2022). These compounds are known to impart a certain ‘velvety and mild astringency’ (Zhang, Cao, et al., 2020). In the low-polyphenol context of ‘Baiye 1′, moderately controlling their levels by adjusting drying conditions may thus be particularly important for achieving a mellow mouthfeel.

### 3.3 Dynamic changes in VOCs during BYBT processing

**Fig. 3:**
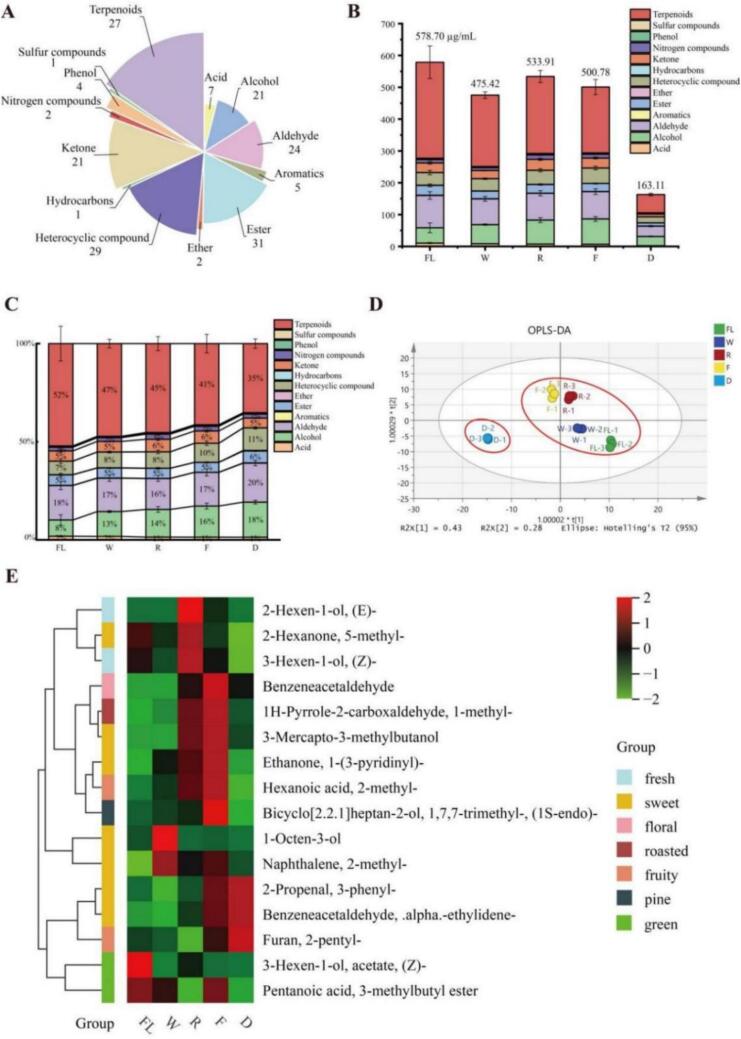
Dynamic changes of VOCs during processing stages of BYBT. (A) Classification of total VOCs with rOAV >1, (B) Changes in the absolute content (μg/kg) of total VOCs during processing, (C) Changes in the relative content (%) of different aroma categories during processing, (D) Dynamic trajectory of VOCs in BYBT, (E) Heatmap of 16 key differential VOCs at the intersection of OPLS-DA (VIP > 1.5) and Random Forest (top 30) screening.

The VOCs in BYBT processing stages were analyzed using HS-SPME-GC/MS. This approach identified 175 volatile compounds with rOAV >1 across BYBT processing stages (Table S1) and classified into 13 subcategories (Fig. 3A). The total volatile concentration, highest in fresh leaves (578.7 μg/mL), significantly decreased to 163.11 μg/mL in dried leaves (Fig. 3B). Relative concentration analysis (Fig. 3C) revealed a decline in terpenes and increases in alcohols, heterocyclic compounds and aldehydes; these four categories collectively accounted for over 84 % of total volatiles by the drying stage, dominating the aroma profile. The decrease in terpenes is likely associated with glycosidic hydrolysis and thermal degradation, while the accumulation of alcohols, aldehydes, and heterocyclic compounds suggests increased contributions from pathways such as enzymatic oxidation, lipid oxidation, and Maillard/Strecker degradation.

**Fig. 4:**
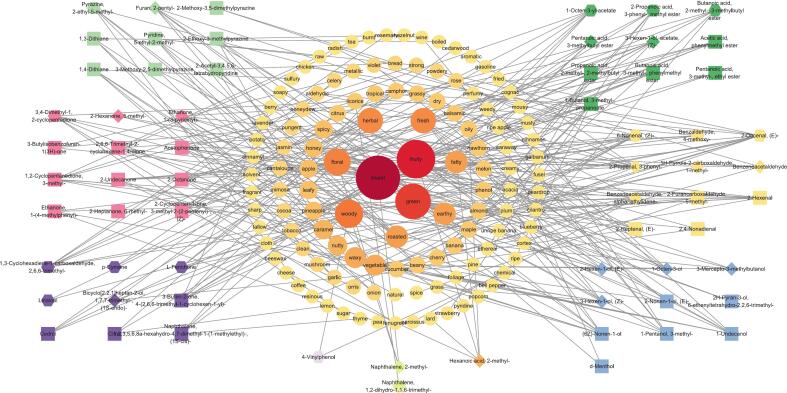
Correlation network between sensory attributes and aroma-related differential metabolites. Note: Sensory attributes are represented by circles, differential metabolites (VIP > 1) by squares, key VOCs identified by RF analysis by hexagons, and overlapping compounds by diamonds. Edges represent metabolite-sensory attribute annotations. Circle size and color intensity correlate with the number of annotated metabolites and the importance of the sensory attribute. Node colors indicate distinct categories.

OPLS-DA analysis effectively distinguished volatile compound profiles across processing stages (R^2^X = 0.936, Q^2^ = 0.929, Fig. 3D), with pronounced differences between enzymatic oxidation stages and thermal oxidation. Permutation tests confirmed model reliability (R^2^ = 0.618, Q^2^ = 0.834, Fig. S2B). Multivariate statistical analysis identified 55 differential volatile metabolites. Separately, Random Forest (RF) analysis, an ensemble learning method (Belgiu & Drăguţ, 2016), pinpointed 30 key volatile compounds. A correlation network analysis was then performed using the union of these two sets of compounds with their literature-derived sensory attributes. This revealed that 22 compounds from this combined set were associated with “sweet” aroma characteristics (Fig. 4), suggesting that “sweet” notes may be a significant component of BYBT's potential aroma profile. This observation aligns with our team's sensory evaluation results for BYBT (Chen et al., 2025) and provides a basis for focusing on these specific volatiles in further studies. A heatmap of the 16 volatile compounds common to both analyses then visualized their dynamic trends across processing stages (Fig. 3E): fresh-aroma alcohols increased during rolling, mushroom flavor 1-Octen-3-ol peaked during withering, and 3-Mercapto-3-methylbutanol associated with roasted/meaty notes, accumulated during the fermentation stage, reflecting distinct formation patterns for different aroma types at specific processing points. Sweet-associated compounds like 3-phenyl-2-propenal (cinnamaldehyde) and α-ethylidenebenzeneacetaldehyde, along with fruity 2-pentylfuran, increased during fermentation and persisted at high levels post-drying, indicating their significant contribution to the final volatile profile.

### 3.4 Formation pathways of VOCs shaping BYBT aroma

Tea aroma biogenesis is a complex interplay of multiple pathways, including lipid oxidation, amino acid degradation (Maillard reaction/Strecker degradation), glycosidic precursor hydrolysis, and carotenoid degradation (Dudareva et al., 2013; Ho et al., 2015). Our results suggest that the unique biochemistry of ‘Baiye 1’ influences the relative contributions of these pathways compared to traditional high-polyphenol black teas (Fig. S3). Fatty acid-derived volatile compounds (FADVs), undergo significant changes. We observed shifts in FADVs like hexanal, 1-octen-3-ol, and trans-2-decanal, consistent with literature reports linking hexanal/1-octen-3-ol to linoleic acid oxidation and others like nonanal/heptanal to oleic/palmitoleic acid oxidation (Wang, Li, et al., 2025). Concurrently, the formation of fruity esters and other aldehydes/alcohols occurs via enzymes like HPL, ADH, and AAT. The high activity of the lipid oxidation pathway in ‘Baiye 1’ may be attributed to its unique biochemical background. This is likely due to two compounding factors: first, the inherently low polyphenol content in this albino cultivar leads to reduced inhibition of lipoxygenase (LOX) enzymes, given their well-documented inhibitory effect (Hong & Yang, 2003; Wang, Huang, et al., 2025). Second, it is possible that ‘Baiye 1’ inherently possesses higher basal LOX activity. Together, these factors promote a robust generation of Fatty Acid-Derived Volatiles (FADVs), which in turn critically shape BYBT's final aroma. These FADVs can contribute desirable fruity or fatty notes but also risk causing green, grassy off-flavors if this pathway is mismanaged during processing. Therefore, developing targeted processing strategies to modulate FADV formation is a key objective for optimizing the quality of albino teas like BYBT. For instance, techniques such as shaking-withering will promote FADV formation in black tea (Zhang, Yu, et al., 2025), while post-harvest treatments like low-temperature or UV-C exposure have shown potential in increasing specific desirable aroma compounds in albino teas (Xie et al., 2025). These examples underscore the potential to refine BYBT's aroma by strategically influencing lipid metabolism and related precursor pathways through tailored processing.

The Maillard reaction and Strecker degradation are likely amplified in BYBT due to its high amino acid content. These pathways, primarily active during the thermal drying stage, generate crucial aroma compounds (Lu et al., 2024). The sustained high level of Phenylacetaldehyde, a key contributor to the honey-like aroma (Maoz, Lewinsohn, and Gonda, 2022), is particularly noteworthy. Its concentration peaked during fermentation, likely due to the enzymatic oxidation of phenylethanol, a pathway common in conventional black teas (Aaqil et al., 2024). However, instead of decreasing due to volatilization during drying, its abundance remained high. This strongly suggests a second, powerful formation mechanism was activated during the thermal stage. We propose this is the Strecker degradation of the exceptionally abundant phenylalanine precursor in ‘Baiye 1’ (Chen et al., 2025). Therefore, the prominent honey-like aroma of BYBT is likely a cumulative result of both pathways, with the thermal reaction during drying playing a uniquely critical role in maintaining the high concentration, a feature directly linked to the cultivar's high-amino-acid biochemistry. The presence of nitrogen-containing heterocyclic compounds like 2-Ethyl-3,5-dimethylpyrazine and 5-Ethyl-2-methylpyridine contributes potential potato or roasted notes, this is consistent with the ‘roasted sweetpotato aroma’ previously observed by our team in BYBT (Chen et al., 2025). 2-Ethyl-3,5-dimethylpyrazine has been reported as key contributors to the roasted aroma in certain albino green tea varieties (Li, Han, et al., 2025). Interestingly, we identified only two Strecker aldehydes among the major VOCs. This contrasts with high-polyphenol black teas where Strecker aldehydes can accumulate excessively (Chen et al., 2021) and might be explained by the lower availability of flavanol oxidation products in ‘Baiye 1’, which can act as catalysts for Strecker degradation (Ho et al., 2015). This potentially leads to a different balance of Maillard/Strecker products in BYBT compared to conventional black teas. These findings suggested that optimizing processing parameters, such as increased rolling intensity, extended fermentation duration, and elevated drying temperature may collectively promote polyphenol oxidation-driven Strecker degradation and amino acid-involved Maillard reactions, resulting in an intensified and refined characteristic aroma profile for BYBT.

**Fig. 5:**
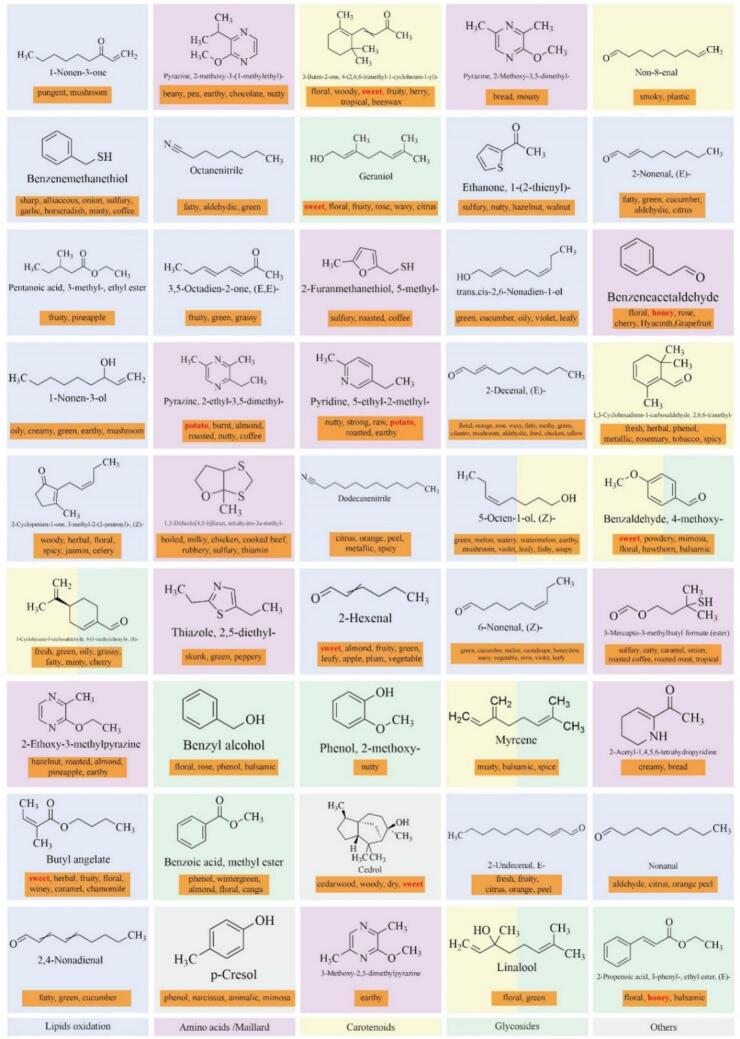
Structural features, nomenclature, flavor characteristics of top 45 VOCs with rOAV >1. Note: The eight key aroma compounds contributing to the characteristic sweet, honey, and roasted sweet potato notes are highlighted in red font. (For interpretation of the references to color in this figure legend, the reader is referred to the web version of this article.)

Hydrolysis of glycosidically bound volatiles serves as another source of aroma, releasing aglycones like terpene alcohols and aromatic alcohols during processing (Song et al., 2018). Geraniol (rose-like, sweet) and (*E*)-Ethyl cinnamate (derived from cinnamic acid released via hydrolysis) were identified as contributors in BYBT. However, the overall contribution from this pathway, particularly key floral notes from linalool and geraniol, might be relatively lower in ‘Baiye 1’ compared to some traditional cultivars (Dong et al., 2018). This could potentially be linked to the altered chloroplast development and associated metabolic pathways (e.g., terpene synthesis) characteristic of albino phenotypes (Zheng et al., 2021). Carotenoid degradation products, such as β-Ionone (floral, woody), also contribute to tea aroma (Ho et al., 2015). While β-Ionone was identified as potentially key in BYBT, the contribution from this pathway might also be limited by the typically lower carotenoid content in albino tea leaves, potentially affecting levels of related compounds like β-damascenone as well (Lu et al., 2019). In contrast, volatiles derived from lipid oxidation and Maillard reactions were predominant among the top 45 high-rOAV compounds (Fig. 5), implying these pathways potentially hold greater significance in ‘Baiye 1’ black tea aroma development, which aligns with its high amino acid and low polyphenol attributes.

### 3.5 Dynamic alteration of lipids during BYBT processing

**Fig. 6:**
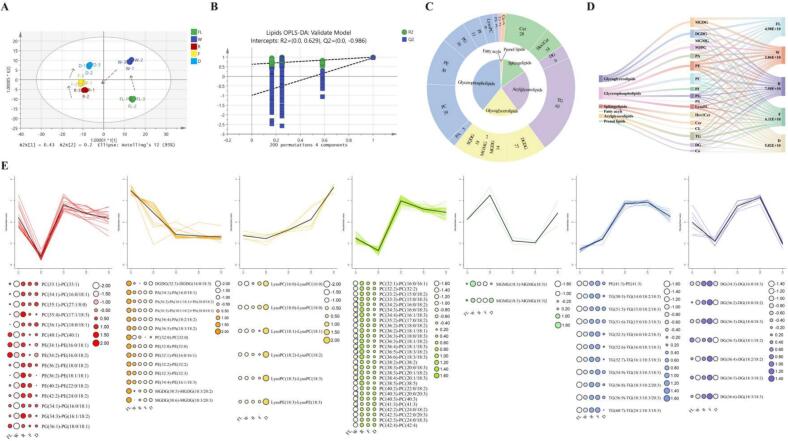
Dynamic changes of lipids in BYBT during different processing stages. (A) Dynamic trajectory of lipid metabolites, (B) 200 repeated cross-validation (R^2^ = 0.629, Q^2^ = −0.986), (C) Secondary pie chart of differential lipid metabolites, (D) Proportions and changes of different lipid subclasses in tea samples, (E) K-means clustering analysis.

During BYBT processing, 296 lipid compounds were identified, spanning six categories and 15 subclasses (Fig. 6C), including 72 acylglycerolipids (AGs), 116 glycerophospholipids (GPLs), and 60 glycoglycerolipids (GLs). To elucidate their dynamic changes, a supervised OPLS-DA model (R^2^X = 0.936, R^2^Y = 0.992, Q^2^ = 0.934, Fig. 6A), validated by permutation tests (Fig. 6B), was established. These results indicated that withering and rolling significantly altered lipid content, while the transformation of lipids into aromatic compounds was accelerated during drying. In contrast, changes in lipid profiles during fermentation were minimal. This highlights that lipid metabolism is particularly active during the initial mechanical processing and later thermal stages, rather than during fermentation. Further K-means clustering of 102 lipid differential metabolites identified seven groups (K = 7), with 38 compounds showing decreased concentrations or dynamic equilibrium after drying, while 64 compounds increased during processing (Fig. 6E).

The observed dynamic patterns of lipid alterations, specifically the extensive degradation of GPLs (e.g., PC, PE, PG, PA) and AGs (e.g., TG) with the concomitant release of FFAs during withering and rolling (Fig. 6D), align well with established lipid hydrolysis phenomena in black tea processing (Zhang et al., 2024). This degradation is significantly influenced by mechanical cell disruption induced by rolling, which is widely recognized as a crucial factor that enhances the interaction between lipolytic enzymes and their substrates, thereby accelerating these hydrolytic processes (Mahanta et al., 1993). Particularly noteworthy in this study is the substantial degradation of chloroplast-specific galactolipids (MGDG and DGDG). This finding is highly consistent with the unique physiology of albino cultivars, which are reported to possess a distinct thylakoid membrane lipid composition (Zhou et al., 2022) and a unique accumulation of nitrogen-containing compounds like phenacetamides (Zheng et al., 2021). The breakdown of these lipids, which are rich in polyunsaturated fatty acids, is therefore a critical step that provides an abundant substrate pool for aroma generation during the processing of ‘Baiye 1’. In BYBT, for instance, the substantial degradation of these PUFA-rich GLs (especially α-linolenic acid, 18:3) during rolling is particularly noteworthy. This process primarily liberates these polyunsaturated fatty acids, which are key precursors for green leaf volatiles (GLVs, e.g., leaf aldehydes/alcohols) and jasmonates, compounds known to contribute characteristic fresh and floral notes to tea (Li et al., 2017). Moreover, carbonyls (e.g., aldehydes, ketones) derived from the oxidation of these FFAs can further react with the abundant amino acids in ‘Baiye 1’ via Maillard reactions and Strecker degradation, generating an even more diverse spectrum of aroma compounds (Shahidi & Hossain, 2022). These cross-reactions between lipid-derived and amino acid-derived metabolites are likely of heightened significance in ‘Baiye 1’, given its high amino acid content and potentially enhanced lipid oxidation, for developing its complex and unique sweet aroma characteristics (such as its reported honey-like and sweet potato-like notes). Given the importance of lipid oxidation, optimizing parameters such as increased kneading intensity, extended fermentation time, and moderately elevated drying temperature may enhance BYBT's flavor characteristics by promoting this reaction.

## 4 Conclusion

This study systematically tracked the dynamic chemical changes in NVOCs, VOCs, and lipids during BYBT processing, elucidating their contributions to its unique flavor profile. Key NVOC alterations included significant amino acid accumulation, contributing to umami and sweetness, and a marked reduction in catechins (including EC, EGCG, EGC, and GC), which led to a mellow taste. Thermal reactions during drying were particularly impactful for NVOCs in BYBT due to its high-amino-acid and low-polyphenol biochemistry. VOC analysis revealed a processing-induced shift towards a profile dominated by alcohols, aldehydes, and heterocyclic compounds. Lipid oxidation and Maillard/Strecker reactions, likely amplified by BYBT's distinct biochemistry, were identified as major aroma formation pathways. Notably, compounds associated with “sweet” notes were prominent, with phenylacetaldehyde (honey-like) and specific pyrazines (linked to roasted sweetpotato characteristics, aligning with sensory data and other albino tea studies) being significant. Extensive lipid degradation (GPLs, GLs, AGs), especially during rolling and withering, released abundant free fatty acid precursors, including linoleic and α-linolenic acid. The subsequent oxidation of these FFAs, and their interactions with amino acid degradation pathways, were crucial for generating a diverse spectrum of aroma compounds. These integrated insights into the flavor mechanisms of this high-amino acid, low-polyphenol specialty tea provide a scientific basis for optimizing ‘Baiye 1’ processing to further enhance its unique quality.

## CRediT authorship contribution statement

**Lin Chen:** Writing – original draft, Software, Investigation, Funding acquisition, Formal analysis, Data curation. **Yuxuan Shi:** Writing – original draft, Software, Investigation, Formal analysis, Data curation. **Jingyi Pan:** Investigation, Data curation. **Yueling Zhao:** Investigation, Data curation. **Liping Liu:** Resources. **Qun Ye:** Investigation. **Yuefei Wang:** Writing – review & editing. **Zhonghua Liu:** Writing – review & editing. **Ping Xu:** Writing – review & editing, Resources, Funding acquisition, Conceptualization.

## Declaration of competing interest

The authors declare that they have no known competing financial interests or personal relationships that could have appeared to influence the work reported in this paper.

## Data Availability

Data will be made available on request.
